# Time from symptoms onset to remdesivir is associated with the risk of ICU admission: a multicentric analyses

**DOI:** 10.1186/s12879-023-08222-y

**Published:** 2023-05-04

**Authors:** Rodrigo Alonso-Navarro, Margarita Ramírez, Mar Masiá, Roger Paredes, Rocío Montejano, Marina Povar-Echeverria, Jordi Carratalà, Miguel Salavert, Enrique Bernal, Carlos Dueñas, Juan Flores, Francisco Fanjul, Isabel Gutiérrez, Verónica Rico, Lourdes Mateu, Julen Cadiñanos, Juan Berenguer, Alex Soriano

**Affiliations:** 1 Department of Infectious Diseases, Clinic Hospital of Barcelona, Barcelona, Spain; 2 Gregorio Marañón General University Hospital (IiSGM), Madrid, Spain; 3 Elche General University Hospital, Elche, Spain; 4 Carlos III Health Institute-CIBERINFEC, Madrid, Spain; 5 German Trias i Pujol University Hospital, Barcelona, Spain; 6 La Paz University Hospital, Madrid, Spain; 7 Miguel Servet University Hospital, Zaragoza, Spain; 8 Bellvitge University Hospital-IDIBELL, University of Barcelona, Barcelona, Spain; 9 La Fe Universitary and Politechnic Hospital of Valencia-IIS-La Fe, Valencia, Spain; 10 Reina Sofía University Hospital of Murcia, Murcia, Spain; 11 Clinic University Hospital of Valladolid, Valladolid, Spain; 12 Arnau de Vilanova University Hospital, Lleida, Spain; 13 Son Espases University Hospital-IdISBa, Palma de Mallorca, Spain

**Keywords:** SARS-CoV-2, Remdesivir, Days from symptoms onset, ICU, Progression

## Abstract

**Background:**

Shorter duration of symptoms before remdesivir has been associated with better outcomes. Our goal was to evaluate variables associated with the need of ICU admission in a cohort of hospitalized patients for COVID-19 under remdesivir including the period from symptoms onset to remdesivir.

**Methods:**

We conducted a retrospective multicentric study analysing all patients admitted with COVID-19 in 9 Spanish hospitals who received treatment with remdesivir in October 2020. The main outcome was the need of ICU admission after 24 h of the first dose of remdesivir.

**Results:**

In our cohort of 497 patients, the median of days from symptom onset to remdesivir was 5 days, and 70 of them (14.1%) were later admitted into ICU. The clinical outcomes associated with ICU admission were days from symptoms onset (5 vs. 6; *p* = 0.023), clinical signs of severe disease (respiratory rate, neutrophil count, ferritin levels and very-high mortality rate in SEIMC-Score) and the use of corticosteroids and anti-inflammatory drugs before ICU. The only variable significatively associated with risk reduction in the Cox-regression analyses was ≤ 5 days from symptoms onset to RDV (HR: 0.54, CI95%: 0.31–0.92; *p* = 0.024).

**Conclusion:**

For patients admitted to the hospital with COVID-19, the prescription of remdesivir within 5 days from symptoms onset diminishes the need of ICU admission.

## Background

From the beginning of the pandemics, the need of an effective antiviral therapy to treat patients infected with SARS-CoV-2 became a major urgency. The clinical trials performed to evaluate the impact of monoclonal antibodies or antiviral agents in patients with mild symptoms have clearly demonstrated that the inhibition of viral replication at early stages of the disease significantly reduce the risk of progression to hospitalization or death [1–5]. In contrast, the impact of different antiviral strategies in patients with moderate to severe disease requiring hospitalization is under debate [6–11]. The evidence from clinical trials suggests that patients under mechanical ventilation do not benefit from antiviral therapy probably because the alveolar damage is so diffuse and cannot be repaired by inhibiting the viral replication. However, in early stages of the disease (with or without oxygen support) the results were controversial. First clinical trials with remdesivir (RDV) showed no mortality reduction [9, 12], but a significant reduction of the time to recovery [7] and the risk of progression among patients not under mechanical ventilation at the moment of randomization have been described by others [9, 13].

As pandemics evolved, the literature regarding the use of RDV has increased exponentially, and most studies suggest that the effectiveness depends on the prior duration of symptoms before receiving RDV [7]. This is in line with reports showing that patients who developed severe pneumonia within the first week of symptoms had the highest viral load and these two circumstances together were associated with the highest mortality rate [14, 15]. Indeed, an antiviral intervention with a potent combination of monoclonal antibodies (casirivimab/imdevimab) showed only a significant reduction in the mortality rate of patients with severe pneumonia among seronegative patients that had a median (IQR) number of days from symptoms onset of 7 (4–10). In contrast, the whole cohort including seropositive patients, in whom no benefit was observed, the median (IQR) days was 9 (6–12), and it would be longer by removing those seronegative [16]. These results indicate that failing to early control viral replication is associated with worse outcome. Interestingly, the final report of both Solidarity and Discovery studies specified that the absence of benefit was only found in patients with an advanced stage of the infection, but unfortunately the impact of early RDV administration was not evaluated [8, 9].

This evidence is consistent to the experience observed for other respiratory viral infections like influenza, where the use of antivirals like oseltamivir significantly reduced the mortality when started within the first 5 days from symptoms onset [17]. However, the information about the impact of the number of days from symptoms onset until the first dose of remdesivir on the efficacy of this antiviral among patients with moderate to severe COVID-19 has not been adequately addressed. Accordingly, the aim of our study was to evaluate the impact of RDV administration on the ICU admission in a large cohort of patients that required hospitalization but were not in the ICU at the moment of starting RDV or within the first 24 h after receiving it.

## Methods

### Study design and outcome

We conducted a retrospective multicentric study in Spain, analyzing the characteristics and evolution of a cohort of patients admitted to hospital for COVID-19 and receiving treatment with RDV for 5 days from October 1st to October 31st 2020. The inclusion criteria were: age 18 years or older, SARS-CoV-2 confirmed infection by RT-PCR of naso-pharyngeal swab sample, and treated with RDV, according to the Spanish Agency of Drugs and Health Products criteria: 1) need of supplemental low-flow oxygen; 2) 7 days or less from symptom onset to prescription; 3) at least two of the following three: respiratory rate of 24 breaths per minute (bpm) or higher, oxygen saturation at ambient air 94% or lower, PaO_2_/FiO_2_ 300 mmHg or lower. The exclusion criteria were: need of mechanical ventilation or extracorporeal membrane oxygenation (ECMO) at the time of prescription of RDV; hypertransaminasemia with ALAT and/or ASAT over 5 times the normal range values; and severe chronic kidney disease (glomerular filtration under 30 mL/min or hemodialysis/peritoneal dialysis), and the admission to ICU within the first 24 h from the first dose of RDV.

The main outcome of the study was the need of admission to the ICU. For this objective we consider that those patients that were admitted to the ICU within the first 24 h from starting RDV should be excluded since the potential beneficial effect of an antiviral requires at least 24 h. The study was approved by the Ethics Committee of the Hospital Clinic (HCB/2021/0571).

### Data collection

All the data from the patients was retrospectively collected from the electronic clinical history and registered in a RedCap© database. The registered variables were demographics (age, sex, ethnic), comorbidities (hypertension, diabetes mellitus, chronic heart failure, asthma, chronic obstructive pulmonary disease [COPD], chronic kidney disease, hepatic cirrhosis, HIV infection, active solid or hematological neoplasia, solid organ transplantation, bone marrow transplantation, conjunctive tissue disease or chronic use of corticosteroids), days from symptoms onset to admission and to RDV prescription, signs and symptoms at admission (dyspnea, respiratory rate and Oxygen saturation), analytical parameters at admission (lymphocyte [L] and neutrophile [N] count, and the ratio of N/L, LDH, C-reactive protein, creatinine, glomerular filtration, ferritin and D-dimer), cycle threshold (Ct) value of the RT-PCR when available, biologic treatment (tocilizumab or baricitinib), systemic corticosteroids, and evolution (ICU admission, need of invasive mechanical ventilation (IMV) and 30-day mortality). A composed SEIMC-Score variable was calculated using data from previous variables (age, sex, dyspnea, Oxygen saturation, glomerular filtration and neutrophile/lymphocyte count) [18].

### Statistical analyses

All categorical variables were described using the percentage and absolute number, and analyzed using a Chi-Squared test, or a Fisher exact text when necessary. As for the continuous variables, they were compared using the median and interquartile range (IQR) values. Continuous variables were included in the analysis directly, except the number of days from symptoms onset to RDV that was evaluated as a continuous variable but also dichotomized by the median. Results using both variables are exposed but the final model shown was using the dichotomized variable since we consider is more informative for clinicians. Univariable analysis was performed to determine the variables associated with ICU admission and those with a *p*-value ≤ 0.1 were included in a multi-variable analysis using a Cox-regression model. Statistical significance was considered when *p*-value was < 0.05.

## Results

From a total of 520 included patients, 23 presented one or more exclusion criteria, leaving a final study population of 497 patients. All the collected variables are depicted in Table 1. The median (IQR) age was 63.4 (52.3–74.4) years old, and 190 patients (38.2%) were women. The main comorbidities were hypertension (222 patients, 44%), diabetes mellitus (139 patients, 28.1%), chronic heart failure (80 patients, 16.2%) and COPD and/or asthma (67 patients, 13.6%). The median (IQR) time from symptom onset to RDV prescription was 5 (3–6) days, and 292 patients (58.9%) received the first dose of RDV within 5 days from the symptoms onset. The Ct value was available in 96 patients, with a median (IQR) value of 20 (16–26). The ICU admission rate > 24 h after the initiation of RDV was 14.1% (70 patients). The overall mortality was 12.9% (64/497).

**Table 1:** Characteristics of patients at admission

Variables	NO ICU ≥ 24 (*N* = 427)	ICU ≥ 24 (*N* = 70)	*P*
Female (%)	165 (38.6)	25 (35.7)	0.692
Median (IQR) of age	62.5 (52-75.6)	67.8 (58.6–72.6)	0.332
Nationality (%)			
* Spanish*	284 (77.8)	43 (66.2)	
* Latin-american*	50 (13.7)	13 (20)	
* Others*	31 (8.5)	9 (13.8)	0.124
Median (IQR) of days of symptoms to RDV	[426] 5 (3–6)	[70] 6 (4–7)	**0.023**
Days of symptoms to RDV < = 5 (%)	259 (60.8)	33 (47.1)	**0.036**
Diabetes mellitus (%)	117 (27.5)	22 (31.4)	0.566
Chronic heart disease (%)	70 (16.4)	10 (14.5)	0.860
Hypertension (%)	187 (43.8)	35 (50)	0.365
Liver cirrhosis (%)	2 (0.5)	1 (1.4)	0.367
Chronic kidney disease (%)	15 (3.5)	0 (0)	0.145
COPD/Asthma (%)	63 (14.9)	4 (5.7)	**0.038**
Solid organ transplantation (%)	1 (0.2)	0 (0)	1.000
Neoplasia (solid o hematological) (%)	28 (6.6)	6 (8.6)	0.608
Stem cell transplamtation (%)	0 (0)	0 (0)	
HIV (%)	6 (1.4)	1 (1.4)	1.000
Inflammatory arthritis/Connective disease (%)	13 (3.1)	3 (4.3)	0.483
Chronic steroid treatment (%)	12 (2.8)	1 (1.4)	0.704
Median (IQR) of RT-PCR Cycle threshold	[86] 20.5 (16.7–26)	[10] 20 (14–26)	0.644
Median (IQR) of breaths per minute	[309] 24 (22–26)	[56] 25 (24–28)	**0.002**
Median (IQR) of Oxygen saturation	[425] 93 (91–94)	[70] 92 (90–94)	0.092
Median (IQR) of C-reactive protein (mg/dL)	[420] 12.6 (6.3–35)	[69] 14.9 (7.6–30.3)	0.285
Median (IQR) of LDH (U/L)	[357] 282 (230–362)	[59] 379 (264–476)	**0.000**
Median (IQR) of AST (UI/L)	[278] 41 (29–56)	[50] 42.5 (28–56)	0.945
Median (IQR) of ALT (UI/L)	[397] 31 (20–48)	[65] 31 (20–58)	0.676
Median (IQR) of neutrophil count (x10^3^/µL)	[420] 4.5 (3.1–6.4)	[69] 5.3 (3.5–8.3)	**0.005**
Median (IQR) of lymphocyte count (x10^3^/µL)	[421] 0.9 (0.6–1.3)	[70] 0.8 (0.6–1.2)	0.184
Median (IQR) of neutrophyl/lymphocyte ratio	[420] 4.6 (2.9–7.9)	[69] 6 (3.8–12.4)	**0.014**
Median (IQR) of ferritine (ng/mL)	[280] 508 (229–916)	[48] 667 (416.5-1076.5)	**0.019**
Median (IQR) of Creatinine (mg/dL)	[422] 0.8 (0.7-1)	[68] 0.9 (0.6–1.1)	0.478
Median (IQR) of D-Dimer (mg/mL)	[386] 267.5 (0.6–632)	[61] 322 (165–800)	0.069
Median (IQR) of SEIMC Score	[427] 6 (4–11)	[70] 7 (5–9)	0.101
Invasive mechanical ventilation (%)	-	42 (60)	**0.000**
Steroids treatment (%)	386 (91.7)	67 (98.5)	**0.045**
Bolus of steroids (> 250 mg) (%)	39 (12.3)	12 (19.4)	0.154
Other anti-inflammatory drugs^a^ (%)	128 (31.8)	34 (49.3)	**0.006**
Mortality probability (SEIMC-Score) (%)			**0.0001**
* Low*	54 (12.6)	5 (7.1)	
* Moderate*	152 (35.6)	15 (21.4)	
* High*	94 (22)	32 (45.7)	
* Very high*	127 (29.7)	18 (25.7)	

^a^ Tocilizumab, baricitinib or anakinra

[ ] Data not available for all patients; the number in brackets indicates the N in which the data was collected

The clinical features significantly associated to ICU admission were, the days from symptoms onset to RDV prescription (5 vs. 6; *p* = 0.023), the respiratory rate in bpm (24 vs. 25; *p* = 0.002), neutrophile count in cells/mL (4.6 vs. 4.5; *p* = 0.005), the ratio N/L (4.6 vs. 6; *p* = 0.014), LDH in IU/mL (272 vs. 379; *p* < 0.001), ferritin in ng/mL (508 vs. 667; *p* = 0.019), the use of systemic corticosteroids before ICU admission (91.7% vs. 98.5%; *p* = 0.045), treatment with biological anti-inflammatory drugs before ICU admission (31.8% vs. 4.3%; *p* = 0.006) and a very-high mortality probability in the SEIMC-Score.

The independent variables selected by the Cox-regression model are shown in Table 2. Only the variable ≤ 5 days from symptoms onset to RDV prescription (HR: 0.54, CI95%: 0.31–0.92; *p* = 0.024) was significantly associated with a risk reduction of being admitted to the ICU. In contrast, increasing LDH (HR: 1.003, CI95%: 1.001–1.005; *p* = 0.001) and C-reactive protein (HR: 1.04, CI95%: 1.01–1.06; *p* = 0.014) serum concentrations were significantly associated with a higher risk of being admitted in the ICU. The analysis using the variable number of days from symptoms onset to RDV prescription as a continuous one did not show any change in the final model (HR: 1.18, CI95%: 1.01–1.38; *p* = 0.04).

**Table 2:** Cox-regression analyses to determine the variables independently associated with ICU admission

Variables^a^	Univariable analysis	Multivariable analysis (*N* = 387; *n* = 57)
	**HR (CI 95%); p**	**HR (CI 95%); p**
Age	1 (0.99–1.02); 0.734	
Female	0.89 (0.55–1.45); 0.640	
Days of symptoms to RDV	1.17 (1.02–1.33); 0.021	
Days of symptoms to RDV ≤ 5	0.61 (0.38–0.97); 0.039	0.54 (0.31–0.92); 0.024
Chronic heart disease	0.83 (0.43–1.63); 0.594	
Hypertension	1.21 (0.76–1.94); 0.423	
Neoplasia (solid o hematological)	1.26 (0.55–2.91); 0.589	
Oxygen saturation	0.97 (0.92–1.01); 0.164	
Netrophil/lymphocye ratio	1.01 (1-1.03); 0.072	
LDH (U/L)	1.004 (1.002–1.006); 0.000	1.003 (1.001–1.005); 0.001
AST (UI/L)	1 (0.99–1.01); 0.711	
C-reactive protein (mg/dL)	1.05 (1.02–1.07); 0.000	1.04 (1.01–1.06); 0.014
D-dimer (ng/mL)	1 (1–1); 0.895	
SEIMC-Score	0.99 (0.95–1.03); 0.581	
Anti-inflammatory drug ≥ 24 h pre-ICU	1.21 (0.74–1.98); 0.442	

^a^ Variables were included as continuous ones. For the multivariable analysis, the dichotomized variable of days from symptoms to RDV was included. The neutrophil/lymphocyte ratio is not included in the multivariate analyses because it is included in the SEIMC-Score variable

## Discussion

Our study, for the first time, demonstrates that early administration of RDV is associated with less probability of ICU admission and we establish a cut-off point of 5 days. This finding was obtained in a large cohort of patients in whom important variables associated with ICU admission were collected. The viral load in infections caused by respiratory viruses peak at symptom onset [19], and the immune system, mediated by type I IFN production, progressively controls viral replication [20]. However, SARS-CoV-2 can evade type I IFN response triggering a dysregulated immune response associated with severe disease [21]. The interval from starting viral replication to dysregulated immune response represents the window of opportunity for an antiviral. Ideally, antivirals should be administered to outpatients with low inflammatory response to reduce the risk of hospitalization [3, 4]. Unfortunately, COVID-19 can progress rapidly within the first week from symptoms onset, particularly in patients with co-morbidities [14, 15]. In this population, we described that the earlier the administration of RDV the greater the beneficial effect on the mortality rate [11] and now the same concept is applicable for ICU admission. Interestingly, C-RP and LDH baseline values were also predictors of ICU admission independently of early RDV administration. The former is a biomarker of the inflammatory response and the second of the alveolar damage and both have been directly associated with COVID-19 progression [22]. This means that the window of opportunity to avoid ICU admission becomes narrower as both the inflammatory response and the alveolar damage increase. This was nicely corroborated in a recent article by Padilla et al. [23] showing that the mortality and the need of mechanical ventilation among hospitalized patients with COVID-19 receiving RDV was significantly associated with the cycle threshold (Ct) value as a marker of the viral load and the value of C-RP. Patients with a Ct < 25 (high viral load) and a C-RP < 38 mg/L (low inflammatory response) were the ones that most benefit from RDV treatment. In contrast, Mehta et al. [24] in a large cohort from India Identified that death was significantly lower in patients with an interval ≤ 9 days vs. >9 days (18.1% vs. 33.7%; *p* = 0.004), but not other shorter intervals. A potential explanation is that these authors did not perform a multivariable analysis considering many other parameters of severity.

Other previous studies have evaluated the impact of the time from symptoms onset to RDV on different outcomes. In the Adaptive Covid-19 Treatment Trial (ACTT-1), the rate ratio for clinical recovery (RDV vs. placebo) was 1.37 (95% CI,1.14–1.64) in those who received RDV within 10 days of symptom onset and 1.20 (95% CI, 0.94–1.52) in those who received RDV after 10 days, but the major benefit was observed among those receiving RDV within the first 6 days from symptoms onset [7], in line with our results. In the study by Goldman et al., hospital discharge rate was higher in patients who had symptoms for < 10 days before receiving the first dose of RDV, than those who had symptoms for 10 days (62% vs. 49%), but there was no analysis of shorter cut-offs [25]. Finally, Wong et al. evaluated the early administration (≤ 2 vs. >2 days) of RDV from hospital admission, not from symptoms onset [26]. The time to clinical improvement (HR: 1.14; 95% CI: 1.01–1.29; *P* = 0.038), and the length of hospital stay (difference: −2.56 days; 95% CI: −4.86 to − 0.26; *P* = 0.029) were shorter in the early group, and they had a lower in-hospital death (HR: 0.58; 95% CI: 0.34–0.99; *P* = 0.045) but the interval from symptoms onset to RDV was not evaluated.

The main limitation of the present study is its retrospective nature. The critical variable for this analysis is the number of days from symptoms onset and this is a difficult to collect variable. However, RDV was under a strict indication protocol that included the number of days from symptoms onset. Accordingly, this variable was present in all the medical records of patients receiving RDV. On the other hand, this is a subjective variable and its precision depends on the patient characteristics and this limitation cannot be avoided. The second limitation is that patients in the present study had ≤ 7 days from symptoms onset and, therefore, it was not possible to evaluate longer intervals. The third limitation is that the viral load measured using Ct was available only in 96 patients and, therefore, it was not included in the analysis.

## Conclusions

The ICU admission is a critical outcome since the number of beds is limited and our results demonstrate that the window of opportunity for RDV to avoid the need of ICU among hospitalized patients is ≤ 5 days. This result is in line with the pathogenesis of this virus, like other respiratory viruses, and encourage physicians to use antivirals in early stages, particularly now that we have oral options to avoid hospitalization. However, COVID-19 evolves rapidly to a severe disease so we have to increase the awareness of general population about the warning symptoms in order not to delay seeking for medical assistance within the window of opportunity for antivirals. In the future, it is necessary to evaluate the impact of receiving RDV or other antivirals in patients with 7 to 10 or > 10 days from symptoms onset.

## Data Availability

The datasets used and/or analysed during the current study are available from the corresponding author on reasonable request.

## Abbreviations

RDV: Remdesivir
COPD: Chronic obstructive pulmonary disease
CT: Cycle threshold
IVM: Invasive mechanical ventilation
